# Insights into gene regulation of the halovirus His2 infecting *Haloarcula hispanica*

**DOI:** 10.1002/mbo3.1016

**Published:** 2020-03-25

**Authors:** Sonny T. M. Lee, Jiun‐Yan Ding, Pei‐Wen Chiang, Mike Dyall‐Smith, Sen‐Lin Tang

**Affiliations:** ^1^ Division of Biology Kansas State University Manhattan Kansas United States; ^2^ Biodiversity Research Center Academia Sinica Taipei Taiwan; ^3^ Computational Biology Group Max‐Planck‐Institute of Biochemistry Martinsried Germany; ^4^ Veterinary Biosciences Faculty of Veterinary and Agricultural Sciences University of Melbourne Parkville Vic. Australia

**Keywords:** gene regulation, haloarchaea, halovirus, infection, microarray, pleolipovirus

## Abstract

Gene expression in *Haloarcula hispanica* cells infected with the gammapleolipovirus His2 was studied using a custom DNA microarray. Total RNA from cells sampled at 0, 1, 2, 3, and 4.5 hr postinfection was reverse‐transcribed into labeled cDNA and hybridized to microarrays, revealing temporal and differential expression in both host and viral genes. His2 gene expression occurred in three main phases (early, middle, and late), and by 4.5 hr p.i. the majority of genes were actively transcribed, including those encoding the major structural proteins. Eighty host genes were differentially regulated ≥twofold postinfection, with most of them predicted to be involved in transport, translation, and metabolism. Differentially expressed host genes could also be grouped into early‐, middle‐, and late‐expressed genes based on the timing of their up‐ and downregulation postinfection. The altered host transcriptional pattern suggests regulation by His2 infection, which may reprogram host metabolism to facilitate its own DNA replication and propagation. This study enhances the characterization of many hypothetical viral genes and provides insights into the interaction between His2 and its host.

## Graphical abstract

Gene expression in Haloarcula hispanica cells infected with the gammapleolipovirus His2 was studied using a custom DNA microarray. Temporal and differential expression in both host and viral genes was observed. This study facilitates the  characterization of many hypothetical viral genes and provides insights into the interaction between His2 and its host.

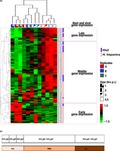

## 1 INTRODUCTION

Viruses infect all three domains of life, and while there is a growing number of studies on virus–host interaction, there is surprisingly little research trying to understand viruses of *Archaea*. Currently, around 110 archaeoviruses have been described, and most have been classified into 17 families and 1 unassigned genus (Krupovic, Cvirkaite‐Krupovic, Iranzo, Prangishvili, & Koonin, 2018; Prangishvili et al., 2017). This compares to more than 6,000 known bacteriophages (Ackermann & Prangishvili, 2012; Pietilä, Demina, Atanasova, Oksanen, & Bamford, 2014), of which more than 3,000 have been formally classified (Munson‐McGee, Snyder, & Young, 2018). Identifying and understanding the interplay of viral and host factors during cell entry, replication, and egress is critical to deciphering the events that determine the fate of infection. The majority of the archaeal viruses isolated so far contain dsDNA as the genetic material and infect halophilic or hyperthermophilic host species (Munson‐McGee et al., 2018; Prangishvili, Forterre, & Garrett, 2006).

*Haloarcula virus* His2 (family *Pleolipoviridae*) infects *Haloarcula hispanica* (Bath, Cukalac, Porter, & Dyall‐Smith, 2006) and is currently the only member of the genus *Gammapleolipovirus* (Krupovic et al., 2018; Pietilä et al., 2012). Virions are pleomorphic and possess a lipid membrane with two exposed spike proteins (VP28, VP29) and two minor membrane‐associated proteins (VP27, VP32) (Pietilä et al., 2012). The virus genome is dsDNA, 16 kb in length with long inverted terminal repeats and terminal proteins, and is predicted to encode a putative type B DNA‐dependent DNA polymerase among its 35 annotated ORFs (Bath et al., 2006). Genome replication is most likely by protein‐priming. At the nucleotide level, His2 shows little similarity to other viruses while at the predicted protein level His2 shows mixed relationships, with the DNA polymerase (His2V_gp14) being similar to that of the spindle‐shaped virus His1 (*Salterprovirus*) while the spike protein (VP29; His2V_gp29) and the AAA ATPase (His2V_gp33) share similarity to the corresponding proteins of betapleovirus HHPV3 (Demina, Atanasova, Pietilä, Oksanen, & Bamford, 2016). 

In single‐step growth studies, virus release begins at around 3 hr postinfection (p.i.) and exit is thought to occur continuously via budding through the cell membrane, as suggested by the retardation of host cell growth concurrent with lipid acquisition by the virus (Bath et al., 2006; Pietilä et al., 2012; Quemin et al., 2016). The lack of cell lysis by His2 (Svirskaite, Oksanen, Daugelavicius, & Bamford, 2016) is a characteristic shared with other haloviruses, such as SH1 (Porter et al., 2005), as well as with members of *Fuselloviridae* such as SSV1 (Fröls, Gordon, Panlilio, Schleper, & Sensen, 2007) and STSV1 (Porter et al., 2005; Xiang et al., 2005). Its mode of replication appears to be very different from the well‐studied lytic infections of model bacterial caudoviruses such as T4 (Desplats & Krisch, 2003), or T3 (Krüger & Schroeder, 1981).

The host species of His2 is the extremely halophilic archaeon *Har. hispanica* (Class Halobacteria*,* family *Haloarculaceae*), which was isolated from a solar saltern in Spain and grows optimally at 25% (w/v) salinity (Juez, Rodriguez‐Valera, Ventosa, & Kushner, 1986). It is an aerobic heterotroph, and like many haloarchaea, the cells of this species have a simple cell envelope consisting of the cell membrane enclosed by a thin, paracrystalline protein layer (S‐layer). The genome sequence of *Har. hispanica* has been determined (Ding, Chiang, Hong, Dyall‐Smith, & Tang, 2014; Liu, Zhenfang, et al., 2011), and methods for genetic manipulation are available (Liu, Han, Han, Liu, Zhou, & Xiang, 2011), making this species an attractive model for studying the dynamics of virus infections in haloarchaea.

In the ongoing struggle between viruses and hosts, host cells develop mechanisms to defend against virus predation while viruses evolve to evade host defenses. One approach to gaining more insight into virus–host interactions is to measure and analyze differential gene expression using the microarray technique. This has been used to study archaeal viruses of *Sulfolobus*, such as the fusellovirus SSV1 (Fröls et al., 2007) and the icosahedral virus STIV (Ortmann et al., 2008), allowing the global surveillance host and virus genes over the infection cycle, and revealing differentially regulated gene expression. In more recent studies of *Sulfolobus* viruses, microarrays were used to examine rudivirus SIRV2 infection (Okutan et al., 2013); the dynamics and interplay between the fusellovirus SSV2, plasmid pSSVi and host genes (Ren, She, & Huang, 2013); and the gene expression in SSV1‐ and SSV2‐lysogens as well as in cells coinfected by both viruses (Fusco, She, Fiorentino, Bartolucci, & Contursi, 2015). One insight from these studies is that SSV1 infection does not induce major changes in host (*Sulfolobus*) gene expression (Fröls et al., 2007; Fusco et al., 2015), which is consistent with the continued (but reduced) growth of the host while the virus is constantly shed.

In this study, we monitored changes in the expression of His2 and *Har. hispanica* genes during the infection cycle using a microarray‐based approach. Temporal expression and differential regulation of both viral and host genes were observed and supported the idea that His2 infection, at least over the first 4.5 hr, has a relatively low impact on host gene expression.

## 2 MATERIALS AND METHODS

### 2.1 Strains and culture conditions

*Haloarcula hispanica* strain N601 is a derivative of *Har. hispanica* ATCC 33960^T^ (Ding et al., 2014; Liu, Zhenfang, et al., 2011). It was cultivated in 23% Modified Growth Medium (MGM: 23% SW, 10M Tris.Cl (pH 7.5), 0.5% yeast extract, 0.2% peptone; Dyall‐Smith, 2009) at 37ºC, agitated constantly at 200 RPM. *Escherichia coli* DH5α, used as a host in cloning PCR products, was cultured in LB‐Miller medium (Sezonov, Joseleau‐Petit, & D'Ari, 2007).

### 2.2 Viral infection

His2 virus stocks were produced by infecting early exponential phase *Har. hispanica* strain N601 cultures (OD_600_ = 0.2) with the virus at a multiplicity of 1:10. For preparing infected‐cell RNA, early exponential phase *Har. hispanica* cells grown in 23% MGM medium at 37°C were collected by centrifugation at 5,000 *g* for 15 min at room temperature, the supernatant discarded and the pellet resuspended in 18% MGM medium containing His2 virus (10^10^ PFU), with multiplicity of infection (MOI) in the ratio of 10^8^:10^9^ (cells:virus). Mixtures were incubated for 15 min at 37°C to enable viral infection, after which the cells were pelleted by centrifugation at 5,000 *g* for 15 min at room temperature and the supernatant discarded. Cells were then washed twice with fresh 18% MGM medium, and the final pellet resuspended in 100 ml of 18% MGM medium and incubated at 37°C with slow shaking (100 rpm). About 1 ml samples were taken at 0 (after the absorption, washing, and collection of the samples), 1, 2, 3, and 4.5 hr p.i. (postinfection) for RNA extraction, and an additional 1 ml samples were taken at the same time to determine the virus titer by plaque assay (Dyall‐Smith, 2009). Cultured samples were frozen in liquid nitrogen until further extraction. We used a T0 reference for this type of analysis, and the advantage is that the cells are identical in every respect except one variable, time. The 15 min infection incubation is short compared with the life cycle of His2, and washes were done at RT. In this way, the comparisons were T1/T0, T2/T0, T3/T0, and T4.5/T0.

### 2.3 Infected host cell RNA extraction

**Figure A1:**
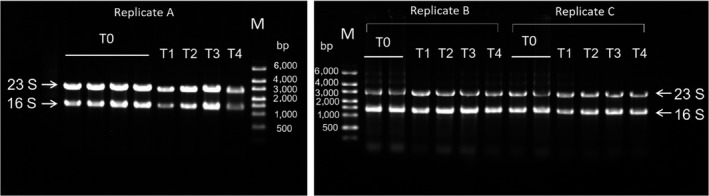
RNA electrophoresis in 1× TBE buffer. The RNA samples were analyzed on 1% agarose guanidine thiocyanate gel stained with ethidium bromide. Electrophoreses were conducted at the voltage 50 V. The arrows indicate the 23S and 16S units of rRNA. T0: after the absorption, washing, and collection of the samples, T1 ~ T4: infection at 1, 2, 3 and 4.5 hr

**Figure A2:**
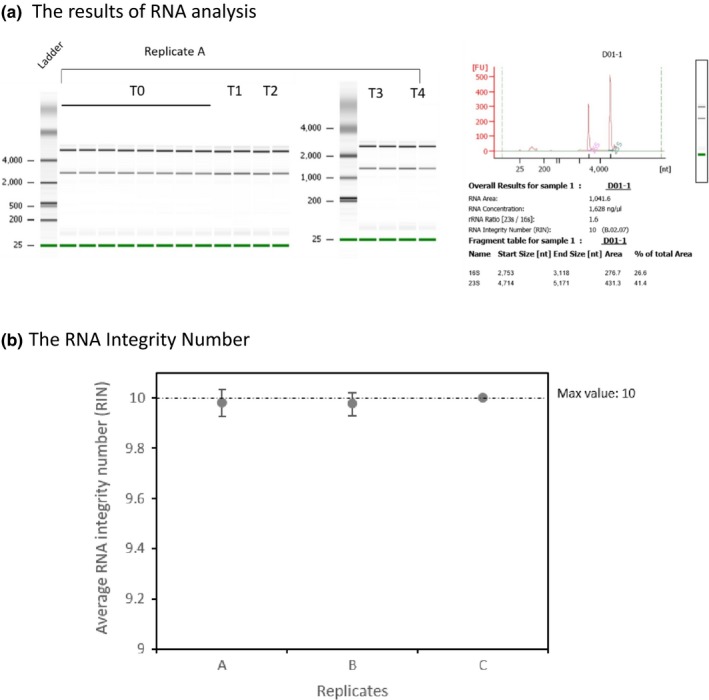
RNA quality and purity by Agilent Bioanalyzer. Panel a is to show one of the gel photographs by using the Bioanalyzer, and its value. All the samples were qualified nearly the best score which is 10 (Panel b).

The TRI‐reagent method (Dyall‐Smith, 2009) was used to extract total RNA from virus‐infected cells. Culture samples (1 ml) were centrifuged at 12,000 *g* (1 min, 4°C), homogenized in 1 ml TRI‐reagent solution (Invitrogen), and incubated at room temperature for 5 min, and then centrifuged at 12,000 *g* (10 min, 4°C). The top (aqueous) layer was transferred to a clean microfuge tube, 200 µl chloroform added, and each mixture vortexed for 15 s, and then incubated at room temperature for 15 min. After incubation, the samples were centrifuged at 12,000 *g* (10 min, 4°C), and the top (aqueous) layer transferred to a clean microfuge tube, 500 µl isopropanol added, and the tubes vortexed for 10 s, and then incubated at room temperature for 10 min. After centrifugation at 12,000 *g* (8 min, 4°C) to pellet RNA, the supernatants were discarded. The RNA pellets were washed twice in 1 ml 75% ethanol and centrifuged at 7,500 *g* for 5 min at 4°C, and then air‐dried before being resuspended in nuclease‐free water. DNase I (BioLabs) was used to remove residual genomic DNA. Briefly, 2 units DNase I and 5 µl of 10× DNase I buffer were added to 5 µg of RNA sample, and incubated at 37°C for 10 min. Following the incubation, we added 0.5 M EDTA (final concentration of 5 mM EDTA) and removed DNase I with Amicon Ultra‐0.5 ml centrifugal filters (Millipore, Ultra 100k) by centrifuging at 6,000 *g* for 6 min at 4°C. The RNA quantity was determined using a NanoDrop ND‐1000 UV‐Vis Spectrophotometer (Nano‐Drop Technologies) and BioAnalyzer 2100 (Agilent 2100 Bioanalyzer) using the Agilent RNA 6000 Nano kit, and RNA integrity was assessed by electrophoresis on 1% agarose‐guanidine thiocyanate gels (Figures A1 and A2).

### 2.4 Microarray design and hybridization

A microarray chip was designed based on the 3,905 annotated genes of *Har. hispanica* strain N601 (BioProject: PRJNA227070). We assayed three biological replicates (A, B, and C) of each sample time using a two‐color platform array and synthesized the complementary DNA from 15 µg total RNA using Superscript^TM^ Plus Indirect cDNA Labeling System (Invitrogen). Reference cDNA samples (0 hr) were synthesized using primers for downstream capture by Cy3, and experimental samples (1, 2, 3, 4.5 hr) were synthesized using primers for downstream capture by Cy5.

We used the Agilent Gene Expression Hybridization Kit for hybridization. Briefly, the microarrays were scanned on an Agilent Technologies Scanner G2505C using the one‐color scan setting for 8 × 15 K array slides. The raw intensity data were then normalized to a global average for each experiment, log_2_ transformed and analyzed using GeneSpring GX7.3.1 (Agilent). A twofold change in gene expression as compared to time 0‐hr was used as the minimum value (or threshold) for describing differences. Z scores were calculated using the log_10_‐transformed gene raw intensity data for each experiment. Z ratio values for each experiment were then calculated by taking the difference between the averages of the observed gene Z scores and dividing by the *SD* of all of the differences for that particular comparison (Cheadle, Vawter, Freed, & Becker, 2003). A onefold change in Z ratio gene expression was used to distinguish significant changes in gene expressions throughout the experiment (Cheadle et al., 2003). Hierarchical cluster analysis (Gene Cluster 3.0) was used to analyze the gene expression profiles between the three replicates. The raw data for all three biological replicates are provided in Table S1 (https://doi.org/10.6084/m9.figshare.11800872).

## 3 RESULTS AND DISCUSSION

### 3.1 Viral infection microarray

**Figure 1:**
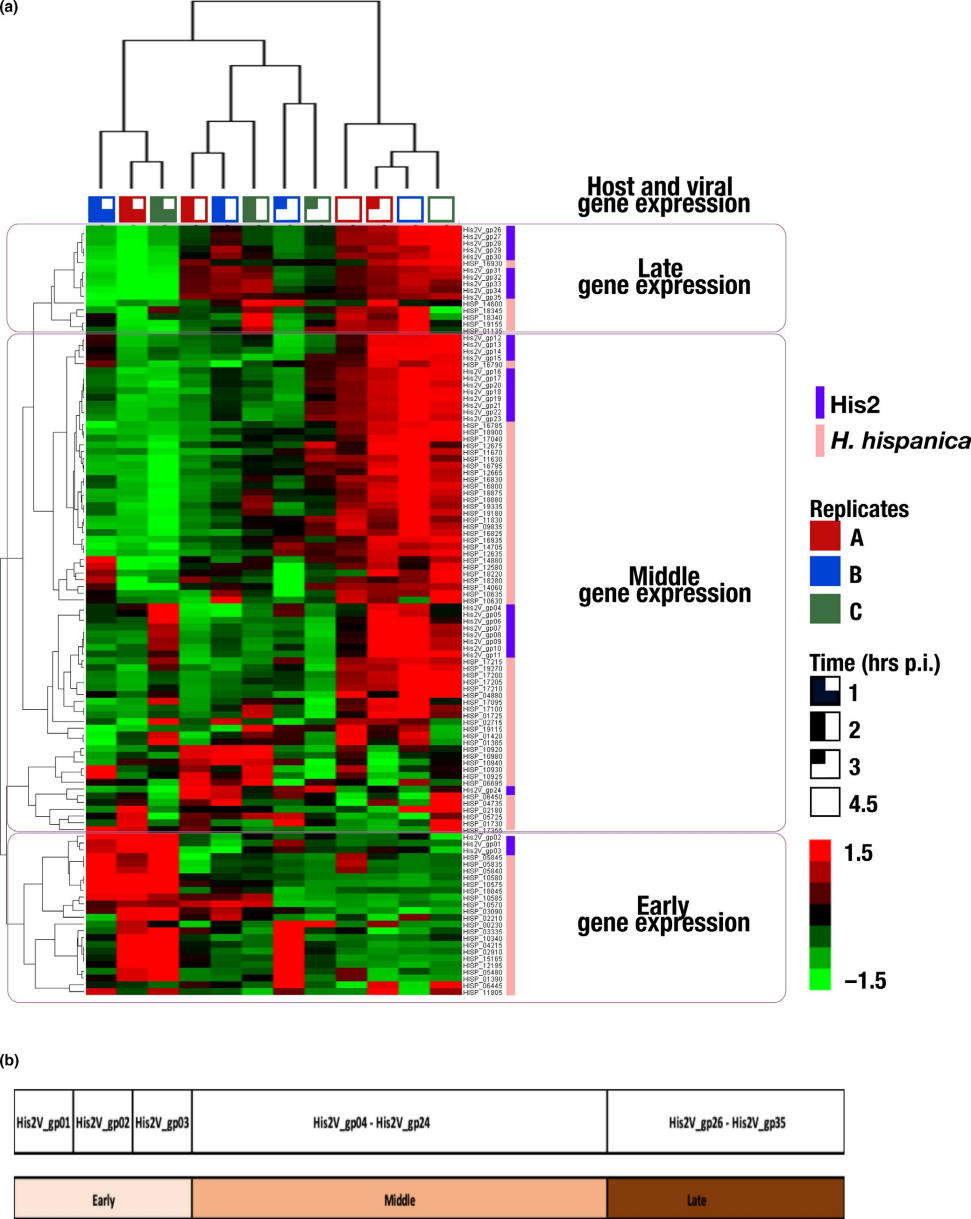
(a) *Haloarcula hispanica* and His2 gene regulation. Heat map showing clustering of viral and host genes across 4‐time points (1, 2, 3, and 4.5) postinfection. The figure represents the mean Z ratio value (*n* = 3) of the transcript ratio of the sample at time *t* = 0 hr. (b) The genome of His2V, with an indication of the early, middle, and late gene expressions

A microarray designed to detect the expression of 3,905 annotated genes of *Har hispanica* strain N601, and 35 genes of halovirus His2 were hybridized to labeled cDNA transcripts of virus‐infected cells sampled from 0 to 4.5 hr postinfection. Three biological replicates were used, and in all cases, virus release was detected at 3 hr p.i. (Table S2; https://doi.org/10.6084/m9.figshare.11800872). The results for genes showing significant regulatory changes have been summarized in Figure 1 and Table 1, and the full compilation of results is given in Table S1A (https://doi.org/10.6084/m9.figshare.11800872). A total of 114 genes (80 genes from the host and 34 from the virus) showing at least a twofold change among the three biological replicates were detected. The heat map shown in Figure 1 provides a graphical summary of the changes in gene expression for both virus and host (indicated at the right edge). The three replicates for each time point are indicated at the top, and group together as expected for the 1 and 2 hr sample times, while one of the 3 hr samples branches with the 4.5 hr group. Hierarchical clustering of genes based on their expression patterns is shown at the left edge of the map and groups genes into three major phases; early, middle, and late. To extend and more confidently substantiate findings reported here, future work should employ high‐replicate designs based on the protocols developed in this study, which will further resolve the understanding of His2 and its host.

**Table 1:** Up‐ and downregulation of *Haloarcula hispanica* and His2 genes (subset; see Table S1 for a full set; https://doi.org/10.6084/m9.figshare.11800872)

	Gene_expression	locus_tag	Process		Gene/ gene product	Sample RNA Z‐score transformation (hours postinfection)			
						1 hr	2 hr	3 hr	4.5 hr
Virus	E	His2V_gp01	‐	‐	hypothetical protein	1.523	−0.789	−0.036	−0.698
	E	His2V_gp02	‐	‐	hypothetical protein	1.586	−0.506	−0.405	−0.674
	E	His2V_gp03	‐	‐	hypothetical protein	1.398	−0.957	0.111	−0.552
	M	His2V_gp24	‐	‐	hypothetical protein	−1.103	0.900	0.707	−0.503
	L	His2V_gp34	‐	‐	hypothetical protein	−1.583	0.805	0.120	0.658
	M	His2V_gp34	‐	‐	hypothetical protein	−1.583	0.805	0.120	0.658
	M	His2V_gp31	‐	‐	hypothetical protein (possible membrane protein, 2TM)	−1.474	0.626	−0.065	0.914
	M	His2V_gp32	‐	‐	hypothetical protein (possible membrane protein, 2TM)	−1.456	0.658	−0.115	0.914
	M	His2V_gp33	‐	‐	hypothetical protein (ATPase‐domain protein)	−1.504	0.776	−0.076	0.804
	M	His2V_gp35	‐	‐	hypothetical protein (possible membrane protein, 1TM)	−1.678	0.592	0.367	0.719
	M	His2V_gp16	‐	‐	hypothetical protein	−1.022	−0.461	0.182	1.300
	M	His2V_gp17	‐	‐	hypothetical protein	−1.005	−0.527	0.214	1.318
	M	His2V_gp18	‐	‐	hypothetical protein	−0.878	−0.633	0.213	1.298
	M	His2V_gp19	‐	‐	hypothetical protein	−0.899	−0.480	0.048	1.331
	M	His2V_gp20	‐	‐	hypothetical protein	−1.084	−0.421	0.289	1.216
	M	His2V_gp21	‐	‐	hypothetical protein	−0.989	−0.573	0.310	1.252
	M	His2V_gp22	‐	‐	hypothetical protein	−0.948	−0.639	0.302	1.284
	M	His2V_gp23	‐	‐	hypothetical protein	−1.008	−0.631	0.386	1.253
	L	His2V_gp26	‐	‐	hypothetical protein (possible membrane protein, 8TM)	−1.153	−0.214	0.031	1.336
	L	His2V_gp27	‐	‐	hypothetical protein (possible membrane protein, 2TM)	−1.179	−0.140	−0.010	1.330
	L	His2V_gp28	‐	‐	hypothetical protein (possible membrane protein, 2TM) (similar TM pattern with VP1)	−1.181	−0.151	−0.028	1.360
	L	His2V_gp29	‐	‐	VP1	−1.322	0.296	−0.013	1.039
	L	His2V_gp30	‐	‐	hypothetical protein (possible membrane protein, 2TM) (similar TM pattern with VP1)	−1.391	0.309	−0.089	1.171
	L	His2V_gp31	‐	‐	hypothetical protein (possible membrane protein, 2TM)	−1.474	0.626	−0.065	0.914
	L	His2V_gp32	‐	‐	hypothetical protein (possible membrane protein, 2TM)	−1.456	0.658	−0.115	0.914
	L	His2V_gp33	‐	‐	hypothetical protein (ATPase‐domain protein)	−1.504	0.776	−0.076	0.804
	L	His2V_gp35	‐	‐	hypothetical protein (possible membrane protein, 1TM)	−1.678	0.592	0.367	0.719
Host	E	HISP_05835	METABOLISM	Inorganic ion transport and metabolism	ABC‐type metal ion transport system, periplasmic component/surface adhesin	1.431	−0.703	−0.642	−0.086
	E	HISP_05840	METABOLISM	Inorganic ion transport and metabolism	ABC‐type Mn/Zn transport systems, ATPase component	1.509	−0.652	−0.653	−0.203
	E	HISP_05845	METABOLISM	Inorganic ion transport and metabolism	ABC‐type Mn2+/Zn2+ transport systems, permease components	1.389	−0.716	−0.557	−0.115
	E	HISP_10570	METABOLISM	Inorganic ion transport and metabolism	ABC‐type phosphate transport system, ATPase component	0.667	1.256	−1.140	−0.783
	E	HISP_10575	METABOLISM	Inorganic ion transport and metabolism	ABC‐type phosphate transport system, permease component	1.584	0.101	−0.800	−0.885
	E	HISP_10580	METABOLISM	Inorganic ion transport and metabolism	ABC‐type phosphate transport system, permease component	1.684	−0.248	−0.655	−0.781
	E	HISP_10585	METABOLISM	Inorganic ion transport and metabolism	ABC‐type phosphate transport system, periplasmic component	1.266	0.648	−1.004	−0.911
	E	HISP_18845	METABOLISM	Energy production and conversion Inorganic ion transport and metabolism	NADH:ubiquinone oxidoreductase subunit 5 (chain L)/Multisubunit Na+/H+ antiporter, MnhA subunit	1.644	−0.254	−0.833	−0.556
	E	HISP_02210	METABOLISM	Energy production and conversion	Archaeal/vacuolar‐type H+‐ATPase subunit B	0.610	0.478	−1.098	0.011
	E	HISP_03090	METABOLISM	Amino acid transport and metabolism Nucleotide transport and metabolism	Carbamoylphosphate synthase large subunit (split gene in MJ)	1.176	0.487	−0.429	−1.234
	E	HISP_17355	METABOLISM	Amino acid transport and metabolism General function prediction only	Histidinol‐phosphatase and related hydrolases of the PHP family	1.003	−0.043	−0.882	−0.077
	M	HISP_10920	INFORMATION STORAGE AND PROCESSING	Translation, ribosomal structure and biogenesis	Ribosomal protein S4E	−0.765	1.438	−0.967	0.294
	M	HISP_10940	INFORMATION STORAGE AND PROCESSING	Translation, ribosomal structure and biogenesis	RNase P/RNase MRP subunit p29	−0.148	1.246	−0.903	−0.195
	M	HISP_10925	INFORMATION STORAGE AND PROCESSING	Translation, ribosomal structure and biogenesis	Ribosomal protein L24	0.271	0.972	−1.104	−0.139
	M	HISP_10930	INFORMATION STORAGE AND PROCESSING	Translation, ribosomal structure and biogenesis	Ribosomal protein L14	0.321	0.683	−1.313	0.309
	M	HISP_10980	INFORMATION STORAGE AND PROCESSING	Translation, ribosomal structure and biogenesis	Ribosomal protein L3	−0.732	1.554	−0.849	0.026
	M	HISP_04735	METABOLISM	Amino acid transport and metabolism	Glutamate synthase domain 2	−0.292	0.572	0.072	−0.352
	M	HISP_05725	METABOLISM	Amino acid transport and metabolism	Choline dehydrogenase and related flavoproteins	−0.050	0.670	0.047	−0.667
	M	HISP_06450	CELLULAR PROCESSES AND SIGNALING	Cell motility Signal transduction mechanisms	Methyl‐accepting chemotaxis protein	−0.507	0.809	0.113	−0.414
	M	HISP_06695	METABOLISM	Amino acid transport and metabolism	3'‐phosphoadenosine 5'‐phosphosulfate sulfotransferase (PAPS reductase)/FAD synthetase and related enzymes	−0.324	1.142	−0.922	0.104
	M	HISP_01730	METABOLISM	Metabolism of cofactors and vitamins	CTP‐dependent riboflavin kinase	0.055	−0.401	0.235	0.111
	M	HISP_04880	METABOLISM	Energy production and conversion	NAD‐dependent aldehyde dehydrogenases	−0.109	−0.687	−0.769	1.566
	M	HISP_09835	METABOLISM	Lipid transport and metabolism	Acyl‐coenzyme A synthetases/AMP‐(fatty) acid ligases	−1.323	−0.339	0.337	1.324
	M	HISP_16785	METABOLISM	Energy production and conversion	Pyruvate/2‐oxoglutarate dehydrogenase complex, dihydrolipoamide dehydrogenase (E3) component, and related enzymes	−1.272	−0.683	0.197	1.224
	M	HISP_16790	METABOLISM	Energy production and conversion	Pyruvate/2‐oxoglutarate dehydrogenase complex, dihydrolipoamide acyltransferase (E2) component, and related enzymes	−0.532	−1.022	0.601	0.953
	L	HISP_16930	METABOLISM	Amino acid transport and metabolism	Selenocysteine synthase [seryl‐tRNASer selenium transferase]	−1.395	0.095	0.419	0.881
	L	HISP_18340	CELLULAR PROCESSES AND SIGNALING	Signal transduction mechanisms	Signal transduction histidine kinase	−0.542	0.160	−0.226	0.608
	L	HISP_19155	METABOLISM	Amino acid transport and metabolism	Glycine cleavage system T protein (aminomethyltransferase)	−0.925	0.132	−0.044	0.837
	M	HISP_02180	METABOLISM	Energy production and conversion	Archaeal/vacuolar‐type H+‐ATPase subunit I	−0.015	0.007	−0.960	0.969
	M	HISP_11670	METABOLISM	Lipid transport and metabolism	Short‐chain fatty acids transporter	−0.975	−0.826	0.814	0.987
	M	HISP_11830	METABOLISM	Carbohydrate transport and metabolism	Phosphoenolpyruvate synthase/pyruvate phosphate dikinase	−1.284	−0.452	0.551	1.184
	M	HISP_12635	METABOLISM	Lipid transport and metabolism	Acyl‐CoA dehydrogenases	−1.171	−0.703	0.737	1.136
	M	HISP_14705	CELLULAR PROCESSES AND SIGNALING	Signal transduction mechanisms	Signal transduction histidine kinase	−1.366	−0.435	0.872	0.929
	M	HISP_16825	METABOLISM	Amino acid transport and metabolism	Aspartate/tyrosine/aromatic aminotransferase	−1.069	−0.725	0.472	1.322
	M	HISP_17040	METABOLISM	Amino acid transport and metabolism	phenylacetate‐CoA oxygenase	−1.015	−0.559	0.363	1.211
	M	HISP_17215	METABOLISM	Inorganic ion transport and metabolism	subunit PaaC	−0.254	−1.024	0.183	1.096
	M	HISP_18900	METABOLISM	Carbohydrate transport and metabolism	ABC‐type sugar transport system, periplasmic component	−1.148	−0.456	0.312	1.291
	M	HISP_19270	METABOLISM	Amino acid transport and metabolism	Phosphoserine phosphatase	−0.402	−0.783	−0.139	1.324

Note

The table shows the stage (early, middle, and late) of gene expression (column—gene expression column) and the peak expression (shaded) Z ratio transcription values postinfection. Z scores were calculated using the log_10_‐transformed gene raw intensity data for each experiment. Z ratio values for each experiment were then calculated by taking the difference between the averages of the observed gene Z scores and dividing by the *SD* of all of the differences for that particular comparison. A onefold change in Z ratio gene expression was used to distinguish significant changes in gene expressions throughout the experiment. Values represent the mean values of three replicates.

### 3.2 Regulation of His2 gene expression

Three phases of gene expression were observed; early (0–1 hr p.i.), middle (2–3 hr p.i.), and late (4.5 hr p.i.). An overview of these phases can be seen in Table 1 (virus, upper panel), where the peak upregulated transcript values for the four sampling times are shaded (blue); and in Table S1B (https://doi.org/10.6084/m9.figshare.11800872) where the up‐ and downregulation of genes are color shaded (blue‐to‐red). In general, early gene expression is focused at the left end of the genome (Figure 1b), then in the middle phase, these are downregulated and genes at the right end are expressed, and by 4.5 hr p.i. (late phase), most genes are strongly expressed (with the conspicuous exception of the early genes, which remain strongly downregulated).

In the early phase, transcription of the first three viral CDS (His2V_gp01, His2V_gp02, His2V_gp03) was observed (Table 1, Table S1B; https://doi.org/10.6084/m9.figshare.11800872), while expression of the other genes was low. These three CDS are short (123–156 nt), closely spaced, and leftwards oriented and are located at the left end of the genome, near the terminal inverted repeat. They encode small proteins (4.5–6 kDa) that show features (pI > 8 and/or CxxC motifs) suggesting they may bind to DNA targets, either of the host or the virus genome (Tarasov, Schwaiger, Furtwängler, Dyall‐Smith, & Oesterhelt, 2011). Since His2 does not encode its own RNA polymerase, transcription of virus genes must use the host RNA polymerase, and early virus gene expression probably utilizes a strong (consensus) haloarchaeal promoter sequence to recruit the host enzyme (Babski et al., 2016). A good candidate sequence, matching the consensus promoter motif SRnnRnnnTTWW (Babski et al., 2016), is found 27 nt upstream (nt 1028–1039) of the start codon of His2_gp03 and could potentially direct the transcription of all three CDS. In well‐studied bacterial viruses, early gene expression commonly includes the production of transcriptional regulators that either suppress host gene expression (Patterson‐West et al., 2018), or facilitate the expression of middle and late genes from virus‐specific promoters (Hinton, 2010; Krüger & Schroeder, 1981). The features of His2 proteins specified by the CDS His2V_gp01‐ gp03) are consistent with these functions.

In the middle phase, the three early phase genes are strongly downregulated and remain so until the last sampling time at 4.5 hr p.i. In contrast, six viral genes (His2V_gp24, His2V_gp31–His2V_gp35) are upregulated at 2 hr p.i. (Table 1, Table S1B; https://doi.org/10.6084/m9.figshare.11800872), with the expression of His2V_gp31–His2V_gp35 remaining upregulated until the late phase. On the viral genome, His2V_gp31–His2V_gp35 are closely spaced, similarly oriented, and located near the right terminal inverted repeat. Most of these five CDS are overlapping and are probably transcribed as a single mRNA. His2V_gp24 encodes a hypothetical protein of unknown function, while His2V_gp31–His2V_gp35 specify two uncharacterized proteins with transmembrane domains (gp31, gp34), a virus structural protein (VP32), an AAA family ATPase (gp33) and a protein with CxxC motifs (gp35) that suggests a role in DNA binding (Nagel, Machulla, Zahn, & Soppa, 2019; Wang et al., 2007).

In the late phase, at 4.5 hr p.i., most genes were upregulated (Table 1, Table S1B; https://doi.org/10.6084/m9.figshare.11800872), including two clusters of consecutive genes; His2V_gp26–His2V_gp35, located near the right end of the genome, and His2V_16–His2V_23. Among the first cluster are genes encoding all four known virion proteins; the two virus spike proteins (VP28 and VP29) and the minor proteins (VP27, VP32) (Pietilä et al., 2012). They also include a potential packaging ATPase (His2V‐gp33). Most of the other six genes specify proteins with membrane domains and their close genomic location and late expression pattern suggest they are also likely to be involved in the assembly of mature (membrane‐containing) virions. The second cluster of upregulated genes are annotated as hypothetical and their functions have not been determined; however, five specify small proteins that carry one or more CxxC motifs suggestive of DNA binding (Nagel et al., 2019).

### 3.3 Host cell gene expression changes during virus infection

Only 80 out of 3,905 host genes (2%) showed significant change (≥twofold) in their expression after His2 infection (Table 1, Table S1C; https://doi.org/10.6084/m9.figshare.11800872). Table 1 shows the times of peak upregulation for these genes, while the color changes in Figure 1, and the shading changes in Table S1, indicate that for many of these genes, their differential expression changed over time from 1 to 4.5 hr p.i. These changes allowed genes to be classified by hierarchical clustering into three phases (early, middle, and late; Figure 1, left and right sides) along with the virus genes.

#### 3.3.1 Early phase host genes

Twenty‐one differentially regulated host genes were designated as early expressed because they were upregulated within the first hour of infection and subsequently downregulated (Figure 1, Table S1C: https://doi.org/10.6084/m9.figshare.11800872.v2). The ten most significantly upregulated early genes are shown in Table 1 (blue shading), and of these, seven specify protein components of two different membrane transport systems; ZnuABC (HISP_05835, 05840, 05845), a specific and high‐affinity Zn^2+^ uptake system (Pederick et al., 2015), and PstABCS (HISP_10570, 10575, 10580, and 10585), a specific (and high‐affinity) importer of phosphate. In bacteria, PstA is not only used for phosphate uptake but is structurally related to PII signal‐transduction proteins and can bind the secondary messenger molecule cyclic‐di‐AMP (c‐di‐AMP), so influencing many different cellular processes (Müller, Hopfner, & Witte, 2015). The presence and significance of c‐di‐AMP in the haloarchaeon *Hfx. volcanii* has recently been described (Braun et al., 2019). Zn^2+^ is not only an important and essential nutrient but it is tempting to speculate that the presence of numerous potential zinc‐finger motifs (CxxC) in many His2 proteins (Nagel et al., 2019), including those encoded by early genes, may be relevant in the upregulation of *znuABC*.

Of the other four genes, two encode membrane‐associated proteins involved in energy production (COG category C); NADH dehydrogenase subunit L (HISP_18845) and V‐type ATP synthase subunit B (HISP_02210). The third gene (HISP_03090) encodes a cytosolic enzyme, carbamoylphosphate synthase, which catalyzes the first committed step in pyrimidine and arginine biosynthesis, and the fourth gene is histidinol‐phosphatase (HISP_17355).

In summary, most of the upregulated genes were involved in the uptake of zinc and phosphate, while the remainder code for proteins with roles in energy production or arginine/nucleotide synthesis. The upregulation of these early genes may reflect the cell responses to membrane damage upon virus entry and/or the effects of early virus proteins that enhance the expression of host genes that favor virus replication.

#### 3.3.2 Middle and late phase host genes (2–4.5 hr p.i.)

A total of 59 differentially regulated host genes were designated as middle‐ or late‐expressed, and as there were only 6 late genes they will be described together with middle genes. These genes were upregulated from 2 to 4.5 hr p.i. (Figure 1, Table S1C: https://doi.org/10.6084/m9.figshare.11800872.v2). Twenty‐seven of the most significantly regulated (middle and late) genes are listed in Table 1 and will be described in more detail. The times of their peak upregulation have been shaded (light blue), and it can be seen that ten genes were significantly upregulated at 2–3 p.i., while the rest peaked at 4.5 hr p.i. (Table 1). Most genes in Table 1 specify proteins that fall into three functional processes (see column 4, Table 1); metabolism (16), information processing (5) and cellular processes (3). At the category level of COG classification (Table S1C,D: https://doi.org/10.6084/m9.figshare.11800872.v2), a number of themes become evident. Firstly, within the metabolism group, four genes specify the protein subunits (E1a, E1b, E2, and E3; COG=C) of pyruvate dehydrogenase (PDH), a multicomplex enzyme that feeds acetyl‐CoA into the TCA cycle and is important in maintaining the supply of energy and biosynthetic building blocks to the cell (Figure 1, Table 1). Other members of the metabolism group include those involved in amino acid metabolism (e.g., HISP_04735, glutamate synthase; COG=E), coenzyme synthesis (e.g., HISP_01730, riboflavin kinase, COG=H), uptake transporters for phosphate/phosphonate (e.g., HISP_17215, COG=P), lipids (e.g., HISP_11670, COG=I), and sugars (HISP_18900, COG=G), as well as genes coding for proteins involved in energy production/conversion (e.g., HISP_02180, V‐type ATPase COG=C).

Among the information processing genes are four that encode ribosomal proteins (S4E, L3, L14, and L24; Table 1; COG=J), which as components of ribosomes participate in protein translation. A fifth gene (HISP_10940) specifies ribonuclease P protein component‐1, an enzyme that is also involved in translation (Table 1, Table S1C, COG=J; https://doi.org/10.6084/m9.figshare.11800872). Interestingly, the hierarchical clustering grouped eight viral genes (His2V_gp16 ‐ His2V_gp23) closely with four host genes (Figure 1, Table 1), suggesting possible regulation by virus gene products. The four host genes included those specifying subunits of PDH (discussed above), an ABC sugar transporter (HISP_18900) and phenylacetate‐CoA oxygenase subunit PaaC (HISP_17040).

Of the two late‐expressed genes shown in Table 1, one encodes a signal transduction histidine kinase (HISP_18340) and the other specifies glycine cleavage system T protein (aminomethyltransferase) (HISP_19155).

Although relatively few host genes showed significant differential regulation during His2 infection, it is likely that the virus has evolved to modify and redirect host cell metabolism in order to optimize virus replication, assembly and exit (Sanchez & Lagunoff, 2015). Other archaeoviruses have previously been shown to have a low impact on host cell metabolism, such as SSV1 (Fröls et al., 2007; Fusco et al., 2015), and in a similar study with the bacterial tectivirus PRD1 and its host *E. coli* (Poranen et al., 2006), changes at the whole‐genome level were described as moderate. In the present study, His2‐infected *Har. hispanica* cells displayed significant upregulation of many genes that are potentially advantageous for the virus, such as pyruvate dehydrogenase (PDH) complex (energy/biosynthesis) and ribosomal proteins (translation). The increased capacity and output of biosynthesis systems could then be redirected into the synthesis of virus components instead of cell growth (Sanchez & Lagunoff, 2015). As many metabolic pathways are inter‐related, the exact mechanisms by which His2 gene products achieve optimal growth of virus be challenging to understand. This study provides a starting point for future investigations aimed at identifying the roles of specific His2 genes in driving metabolic changes in the host, such as the roles of the many small, zinc‐finger motif proteins.

## 4 CONCLUSION

The synchronization of His2 infection of *Har. hispanica* allowed temporal and differential regulation of viral and host genes to be examined. Eighty host genes were differentially regulated ≥twofold postinfection. Both viral and host genes could be grouped into early‐, middle‐, and late‐expressed genes, according to the times at which their transcripts were upregulated. Infection, replication, and propagation of His2 coincided with the regulation of host genes that were involved in transport, energy production, translation, and metabolism. Further studies will be needed to unravel and better understand the virus transcription program and the roles of individual genes in the interplay and evolution of His2 and its host.

## CONFLICT OF INTEREST

None declared.

## AUTHOR CONTRIBUTIONS

Sonny Lee equally contributed to data curation, formal analysis, investigation, methodology, project administration, resources, validation, visualization, writing–original draft, and writing–review and editing; Jiun‐Yan Ding equally contributed to data curation, formal analysis, investigation, methodology, validation, visualization, and writing–review and editing; Pei‐Wen Chiang equally contributed to data curation, formal analysis, investigation, methodology, validation, and visualization; Mike Dyall‐Smith equally contributed to data curation, formal analysis, methodology, resources, validation, and writing–review and editing; Sen‐Lin Tang equally contributed to conceptualization, data curation, funding acquisition, investigation, methodology, project administration, resources, supervision, validation, and writing–review and editing.

## ETHICS STATEMENT

None required.

## Data Availability

All data generated or analyzed during this study are included in this published article and the appendices, as well as Tables S1 and S2 available at https://doi.org/10.6084/m9.figshare.11800872
